# Outcomes after ticagrelor versus clopidogrel treatment in end-stage renal disease patients with acute myocardial infarction: a nationwide cohort study

**DOI:** 10.1038/s41598-021-00360-0

**Published:** 2021-10-21

**Authors:** Ying-Chang Tung, Chi-Jen Chang, Jia-Rou Liu, Shu-Hao Chang, Yi-Hsin Chan, Chi-Tai Kuo, Lai-Chu See

**Affiliations:** 1 Cardiovascular Department, Linkou Chang Gung Memorial Hospital, Taoyüan, Taiwan; 2 College of Medicine, Chang Gung University, Taoyüan, Taiwan; 3 Department of Public Health, College of Medicine, Chang Gung University, 259, Wenhua 1st Rd., Guishan Dist., Taoyüan City, 333 Taiwan (R.O.C.); 4 Biostatistics Core Laboratory, Molecular Medicine Research Center, Chang Gung University, Taoyüan, Taiwan; 5 Division of Rheumatology, Allergy and Immunology, Department of Internal Medicine, Linkou Chang Gung Memorial Hospital, Taoyüan, Taiwan

**Keywords:** Cardiovascular biology, Interventional cardiology

## Abstract

Clinical outcomes are unknown after ticagrelor treatment in patients with end-stage renal disease (ESRD) who are diagnosed with acute myocardial infarction (AMI). ESRD patients who were on hemodialysis and received dual antiplatelet therapy (DAPT) for AMI between July 2013 and December 2016 were identified in Taiwan's National Health Insurance Research Database. Using stabilized inverse probability of treatment weighting, patients receiving aspirin plus ticagrelor (n = 530) were compared with those receiving aspirin plus clopidogrel (n = 2462) for the primary efficacy endpoint, a composite of all-cause death, nonfatal myocardial infarction, or nonfatal stroke, and bleeding, defined according to the Bleeding Academic Research Consortium. Study outcomes were compared between the two groups using Cox proportional hazards model or competing risk model for the hazard ratio or subdistribution hazard ratio (SHR). During 9 months of follow-up, ticagrelor was comparable to clopidogrel with respect to the risks of primary efficacy endpoint [11.69 vs. 9.28/100 patient-months; SHR, 1.16; 95% confidence interval (CI) 0.97–1.4] and bleeding (5.55 vs. 4.36/100 patient-months; SHR 1.14; 95% CI 0.88–1.47). In conclusion, among hemodialysis patients receiving DAPT for AMI, ticagrelor was comparable to clopidogrel with regard to the composite efficacy endpoint and bleeding.

## Introduction

Chronic kidney disease (CKD) has long been considered a major risk factor for cardiovascular disease, and the risk increases linearly as renal function deteriorates^1,2^. CKD and end-stage renal disease (ESRD) not only accelerate the development of coronary artery disease (CAD) but also affect its clinical manifestation and symptoms, with acute myocardial infarction (AMI) more frequently being the initial presentation than stable angina^3,4^. AMI is a catastrophic clinical event for dialysis patients, and outcomes remain bleak even after coronary revascularization^5,6^. The complex hemostatic features of ESRD contribute to a simultaneous prothrombotic milieu and high bleeding risk in this population, which poses a great challenge for clinicians when prescribing dual antiplatelet therapy (DAPT) for ESRD patients who are diagnosed with AMI or who receive coronary stenting. Furthermore, ESRD is often excluded from randomized controlled trials of antithrombotic agents that establish risk–benefit profiles for the general population, precluding extrapolation of these results to this high-risk cohort.

Clopidogrel is the most widely used P2Y_12_ inhibitor and has several shortcomings, including delayed onset of action, modest and variable platelet inhibition, and a high on-treatment platelet reactivity (HPR) for a substantial portion of patients^7–9^. Abundant evidence has indicated an association between HPR under clopidogrel treatment and an increase in cardiovascular events after percutaneous coronary intervention (PCI), including stent thrombosis^10–12^. The prevalence of clopidogrel-related HPR is particularly high in patients with acute coronary syndrome or CKD, which has led to debate about its benefit to these patients^13–16^. Ticagrelor, a potent P2Y_12_ antagonist, provides more rapid onset and offset of action than clopidogrel and depends minimally on kidney function for metabolism and excretion^17^. In the PLATelet inhibition and patient Outcomes (PLATO) trial, ticagrelor, compared with clopidogrel, reduced the composite endpoint of cardiovascular death, nonfatal MI, or stroke but increased the rate of bleeding unrelated to procedure^18^. In a prespecified substudy of the PLATO trial, the advantage of ticagrelor was even greater in patients with CKD, with a 23% relative reduction in the primary ischemic endpoint^19^. However, ESRD was an exclusion criterion of the PLATO trial. Whether ticagrelor is superior to clopidogrel in the ESRD population is unknown. In this study, we used the National Health Insurance Research Database (NHIRD) of Taiwan to compare the efficacy and safety of ticagrelor versus clopidogrel in the treatment of AMI among patients with ESRD requiring maintenance hemodialysis.

## Methods

### Data source

Taiwan's National Health Insurance (NHI) Program is a government-run, mandatory health insurance program, covering approximately 99.9% of citizens in Taiwan^20^. Taiwan's NHIRD, one of the largest administrative healthcare databases in the world, provides patient-level data on basic demographic information, disease diagnosis, prescriptions, operations, investigations, and each outpatient visit or inpatient care details. Previous studies have validated the accuracy of the NHIRD with regard to diagnoses of MI and stroke as well as mortality associated with these events^21–23^. After passing rigid expert review on rationale and privacy protection, the datasets are made available at the National Health Insurance Administration, Ministry of Health and Welfare, Taiwan. The current study was conducted in compliance with the standards of the 1964 Declaration of Helsinki. The Chang Gung Medical Foundation Institutional Review Board approved this study and waived the need of informed consent because patient information had been delinked in the NHIRD (No. 104-2932B).

### Study population and exposure

**Figure 1:**
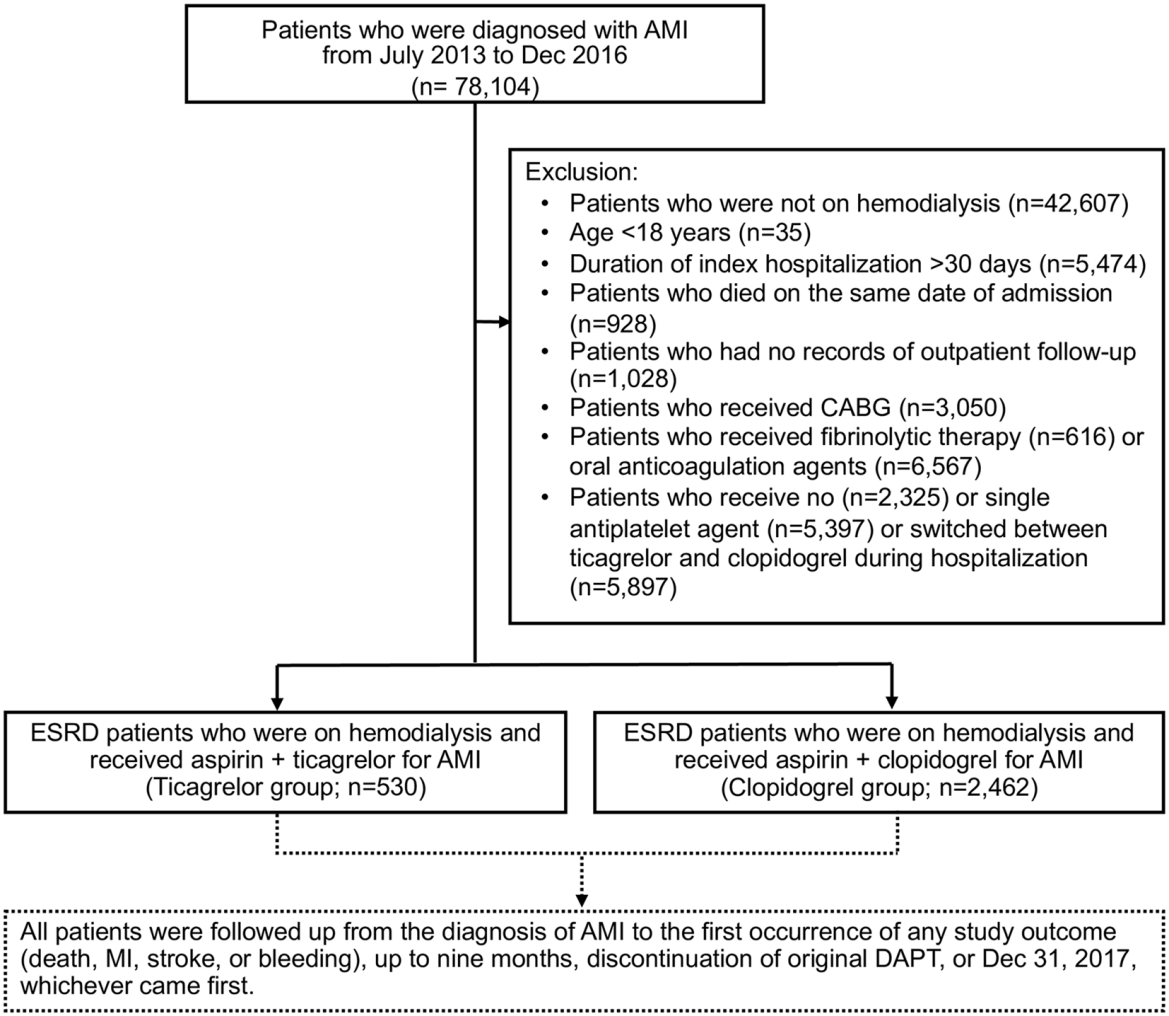
Patient enrollment. *AMI* acute myocardial infarction, *CABG* coronary-artery bypass grafting, *DAPT* dual antiplatelet therapy, *ESRD* end-stage renal disease.

ESRD patients who were on maintenance hemodialysis and admitted with a principal diagnosis of AMI from July 2013 to December 2016 were identified in the NHIRD. This period was chosen because Taiwan's NHI program began to reimburse expenses for ticagrelor starting in July 2013. The diagnoses of ESRD and maintenance hemodialysis, defined at continuous hemodialysis for at least 3 months, were made by the catastrophic illness certificate issued by Taiwan's NHI program. AMI was identified according to the International Classification of Diseases, 9th Revision, Clinical Modification (ICD-9-CM) diagnosis codes 410.x (July 1, 2013 to December 31, 2014) and ICD-10-CM diagnosis codes I21.x and I22.x (January 1, 2015 to December 31, 2015). Figure 1 illustrates patient enrollment. We excluded patients who did not have ESRD or required maintenance hemodialysis (n = 35,497). We also excluded patients who were less than 18 years of age (n = 35); had a duration of index hospitalization longer than 30 days (n = 5474); died on the same date of AMI admission (n = 928); had no records of outpatient follow-up (n = 1028); underwent coronary-artery bypass grafting (CABG; n = 3050); received fibrinolytic therapy (n = 616) or oral anticoagulation agents (n = 6567); and those who received no (n = 2325) or single antiplatelet agent (n = 5397) or switched between ticagrelor and clopidogrel (n = 5897) during hospitalization for AMI. A total of 2992 ESRD patients who were hospitalized for AMI and treated with DAPT were analyzed in this study. The patients who received aspirin and ticagrelor were defined as the ticagrelor group; the remaining patients who received aspirin and clopidogrel were defined as the clopidogrel group. The admission date for AMI was defined as the index date. Some patients switched between P2Y_12_ inhibitors or downgraded DAPT to single antiplatelet therapy for unknown reasons; therefore, any discontinuation of the original DAPT that was not related to clinical events was defined as an endpoint apart from clinical outcomes. All patients were followed up for 9 months or until the original DAPT was discontinued or clinical endpoints were reached, whichever came first. A 9-month follow-up was chosen because Taiwan's NHI program covers ticagrelor or clopidogrel treatment for 9 months, and we could not identify patients who paid for these drugs at their own expense beyond this period.

### Covariates

Reimbursement claims for emergency, outpatient, and inpatient services were used to obtain patient demographics and clinical characteristics. A comorbid condition was defined as a discharge diagnosis or a diagnosis that was confirmed by two or more outpatient visits, based on the ICD-9-CM (before December 31, 2014) and ICD-10-CM diagnosis codes (after January 1, 2015) (Supplemental Table 1). Baseline medication use was defined as medications prescribed during hospitalization for AMI or within half a year before the index date. The use of medical devices and performance of interventional procedures were identified based on the ICD-9-CM and ICD-10-CM procedure codes or the Taiwan NHI reimbursement codes.

**Table 1:** Baseline characteristics of end-stage renal disease patients with acute myocardial infarction treated with aspirin plus ticagrelor versus aspirin plus clopidogrel.

	Before weighting			After weighting		
	Ticagrelor (n = 530) (%)	Clopidogrel (n = 2462) (%)	Standard mean difference	Ticagrelor (n = 530) (%)	Clopidogrel (n = 2462) (%)	Standard mean difference
Age > 75 years	24.90	29.90	− 0.111	28.40	29.00	− 0.014
Male	57.90	57.40	0.011	56.90	57.40	− 0.011
Diabetes mellitus	75.80	74.70	0.028	76.30	74.90	0.034
Hypertension	89.20	90.00	− 0.025	90.00	90.00	0.002
Hyperlipidemia	52.10	55.90	− 0.077	54.40	55.40	− 0.020
Congestive heart failure	51.50	53.10	− 0.032	51.30	52.70	− 0.028
Peripheral artery disease	23.00	28.50	− 0.125	26.50	27.60	− 0.025
Gout	18.90	20.90	− 0.050	19.70	20.60	− 0.021
Atrial fibrillation	10.40	9.91	0.016	9.72	9.93	− 0.007
Previous myocardial infarction	8.49	11.90	− 0.114	10.50	11.40	− 0.030
Previous revascularization	27.20	29.80	− 0.059	29.30	29.40	− 0.002
Previous stroke	10.90	9.34	0.053	8.58	9.55	− 0.034
Previous bleeding	11.50	16.10	− 0.134	14.60	15.30	− 0.022
*Diagnosis of AMI*			0.235			− 0.006
NSTEMI	81.70	89.85		88.80	88.60	
STEMI	18.30	10.15		11.20	11.40	
**Medications and management during hospitalization for AMI**						
Unfractionated heparin	72.80	70.90	0.043	72.00	71.30	0.015
Enoxaparin	19.40	18.20	0.033	18.90	18.40	0.013
GP IIb/IIIa inhibitors	4.91	2.52	0.126	2.85	2.85	0.000
ACE inhibitors	36.80	29.70	0.152	31.10	30.90	0.006
Angiotensin II receptor blockers	33.20	33.50	− 0.006	33.90	33.40	0.011
Beta-blockers	55.30	43.70	0.232	45.80	45.60	0.004
Statins	58.70	48.30	0.208	50.90	50.00	0.017
Proton-pump inhibitors	16.20	18.70	− 0.065	17.60	18.30	− 0.019
*PCI*			0.163			0.029
Bare metal stent	30.75	27.62		28.93	28.21	
Drug-eluting stent	25.47	21.57		22.76	22.23	
POBA	10.38	9.71		9.90	9.75	
None	33.40	41.10		38.41	39.82	

*ACE* angiotensin-converting enzyme, *AMI* acute myocardial infarction, *GP* glycoprotein, *NSTEMI* non–ST-segment elevation myocardial infarction, *POBA* plain old balloon angioplasty, *STEMI* ST-segment elevation myocardial infarction.

### Outcome measures

The primary efficacy endpoint was defined as a composite of all-cause death, nonfatal MI, or nonfatal stroke at 9-month follow-up. The secondary efficacy endpoints included the individual components of the primary efficacy endpoint, with stroke further classified into ischemic and hemorrhagic. The safety endpoint was defined as Bleeding Academic Research Consortium (BARC) type 2, 3, or 5 bleeding^24^. The diagnostic codes of the outcomes are listed in Supplemental Table 1. We excluded BARC type 1 bleeding (bleeding that is not actionable and does not require evaluation or treatment from a healthcare professional) because it could not be captured in the NHIRD. BARC type 4 (CABG-related) bleeding was also excluded according to the study design. Therefore, any bleeding extracted from the database would be BARC type 2, 3, or 5 bleeding. Since the database does not contain hemoglobin data, we modified the definition of BARC type 3 bleeding as bleeding requiring blood transfusion, intravenous vasoactive agents, or exploratory laparotomy; cardiac tamponade; intracranial hemorrhage; intraocular bleeding. Blood transfusion, intravenous vasoactive agents, and exploratory laparotomy were identified with the use of drug codes and procedure codes of the NHIRD. BARC type 5 bleeding was defined as bleeding being the principal diagnosis of admission with mortality within 7 days. BARC type 2 bleeding was defined as bleeding that did not fit the criteria for type 3 or 5 bleeding in this study. For the primary efficacy endpoint (a composite of all-cause death, nonfatal MI, or nonfatal stroke) and the safety endpoint (BARC type 2, 3, or 5), the numerator of the rate was the first event of a study outcome. All clinical outcomes had to be a discharge diagnosis to avoid misclassification. The follow-up period was from the index date to the first occurrence of any study outcome, up to 9 months, or discontinuation of DAPT, whichever came first.

### Statistical analysis

Stabilized inverse probability of treatment weighting (IPTW) was used to balance baseline differences between the ticagrelor and clopidogrel groups^25^. The advantage of stabilized IPTW is that it provides an appropriate estimation of the variance of main effect and maintains an appropriate type I error by preserving the sample size of the original data. The propensity score, defined as the probability of a patient to receive ticagrelor for AMI treatment, was calculated using a generalized boosted model (GBM)^26^ that included all the covariates listed in Table 1. The GBM method gives the best performance in various scenarios (the model is additivity and linearity, mild non-additivity and nonlinearity, and moderate non-additivity and nonlinearity), and various weight trimming percentiles (from 50 to 100)^27^. A standardized mean difference of less than the absolute value of 0.1 was considered to be a negligible difference between the two groups after stabilized IPTW^28^. The incidence rates of clinical endpoints were expressed as the total number of events during the follow-up period divided by the person-months at risk. The risks of all-cause death were compared using the Cox proportional hazards model, with the clopidogrel group as the reference group. To account for the competing risk of death, the incidence rates of nonfatal, time-to-event outcomes (i.e., nonfatal MI, nonfatal stroke, and bleeding) were compared using the Fine and Gray subdistribution hazard model^29^. A *p* value of less than 0.05 was considered statistically significant. All statistical analyses were performed using SAS version 9.4 (SAS Institute Inc., Cary, NC).

### Ethics declarations

The Institutional Review Board of Chang Gung Medical Foundation approved this study (No. 104-2932B).

## Results

From July 2013 to December 2016, a total of 2992 patients with ESRD on maintenance hemodialysis who were diagnosed with AMI were eligible for this study. Among these patients, 530 (17.7%) received ticagrelor and 2462 (82.3%) received clopidogrel (Table 1). Before stabilized IPTW, ticagrelor group patients were younger; more frequently presented with ST-elevation myocardial infarction (STEMI); received PCI or coronary stenting; and were treated with glycoprotein IIb/IIIa inhibitors, angiotensin-converting enzyme inhibitors, beta-blockers, and statins during hospitalization, whereas clopidogrel group patients were older and had higher rates of non-STEMI (NSTEMI), peripheral artery disease, and previous MI and bleeding events. There was no significant difference between the two treatment groups for the following factors: gender; underlying diseases, including diabetes, hypertension, dyslipidemia, heart failure, atrial fibrillation, or previous stroke; or the use of medications, including unfractionated or low-molecular-weight heparin, angiotensin II receptor blockers, or proton pump inhibitors. After stabilized IPTW, the two groups were well balanced for baseline characteristics.

Table 2 shows clinical outcomes after stabilized IPTW. During 9 months of follow-up, there was no significant difference in the incidence rates of primary efficacy endpoint between the two groups (ticagrelor vs. clopidogrel, 11.69 vs. 9.28 per 100 patient-months; subdistribution hazard ratio (SHR), 1.16; 95% confidence interval (CI), 0.97–1.4; *p* = 0.11). Between the two groups, incidence rates were comparable of all-cause death (9.31 vs. 7.22 per 100 patient-months; hazard ratio (HR), 1.17; 95% CI, 0.97–1.42; *p* = 0.11) and MI (1.58 vs. 1.50 per 100 patient-months; SHR, 0.96; 96% CI, 0.61–1.52; *p* = 0.86). Ticagrelor reduced the rate of stroke (0.19 vs. 0.67 per 100 patient-months; SHR, 0.25; 95% CI, 0.07–0.85; *p* = 0.03) after accounting for the competing risk of death, although the numbers of stroke events were small in both groups (4 and 55 in the ticagrelor and the clopidogrel groups, respectively).

**Table 2:** Clinical outcomes of end-stage renal disease patients with acute myocardial infarction treated with ticagrelor versus clopidogrel after stabilized inverse probability of treatment weighting.

Endpoints	Ticagrelor (n = 530)		Clopidogrel (n = 2462)		Cox proportional hazard model		Competing risk analysis	
	Events	Incidence (per 100 PMs)	Events	Incidence (per 100 PMs)	HR (95% CI)	*p* value	SHR (95% CI)	*p* value
**Primary efficacy endpoint**^**a**^	143.4	11.69	689.3	9.28	1.16 (0.97–1.39)	0.11	1.16 (0.97–1.40)	0.11
All-cause death	129.5	9.31	603.1	7.22	1.17 (0.97–1.42)	0.11		
Nonfatal myocardial infarction	21.3	1.58	120.3	1.50	1.05 (0.66–1.66)	0.84	0.96 (0.61–1.52)	0.86
Nonfatal stroke	2.6	0.19	55.7	0.67	0.27 (0.08–0.92)	0.04	0.25 (0.07–0.85)	0.03
**Any bleeding**	68.2	5.55	323.5	4.36	1.25 (0.96–1.63)	0.09	1.14 (0.88–1.47)	0.33
BARC type 2 bleeding	55.9	4.55	248	3.34	1.35 (1.01–1.81)	0.04	1.24 (0.93–1.65)	0.15
BARC type 3 or 5 bleeding	12.3	1.00	75.5	1.02	0.93 (0.51–1.70)	0.82	0.86 (0.47–1.58)	0.63

*BARC* Bleeding Academic Research Consortium, *PM* person-month, *CI* confidence interval, *HR* hazard ratio, *SHR* subdistribution hazard ratio.

^a^Primary efficacy endpoint: a composite of all-cause death, myocardial infarction, or stroke.

With regard to safety outcomes, no significant difference was noted between the two groups regarding the risk of overall bleeding (5.55 vs. 4.36 per 100 patient-months; SHR, 1.14; 95% CI, 0.88–1.47; *p* = 0.33). Ticagrelor increased the rate of BARC type 2 bleeding compared with clopidogrel (4.55 vs. 3.34 per 100 patient-months; HR, 1.35; 95% CI, 1.01–1.81; *p* = 0.04), but the difference was no longer significant after taking the competing risk of death into account (SHR, 1.24; 95% CI, 0.93–1.65; *p* = 0.15). BARC type 3 or 5 bleeding risks were comparable between the two groups (1.0 vs. 1.02 per 100 patient-months; SHR, 0.86; 95% CI, 0.47–1.58; *p* = 0.63).

**Figure 2:**
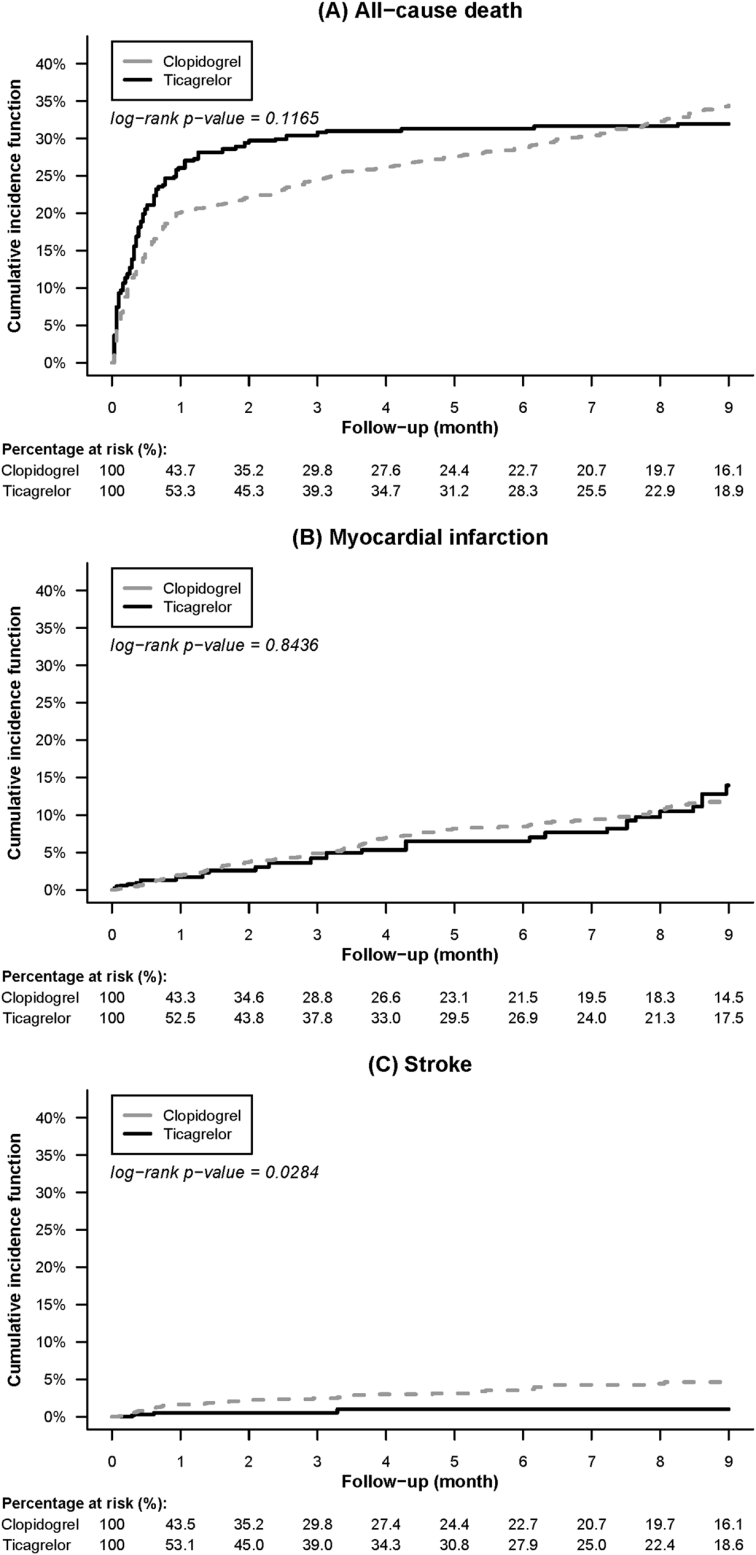
Comparison of the cumulative incidence rates of (**A**) all-cause death, (**B**) nonfatal myocardial infarction, and (**C**) nonfatal stroke among end-stage renal disease patients with acute myocardial infarction treated between the ticagrelor and clopidogrel groups after stabilized inverse probability of treatment weighting.

**Figure 3:**
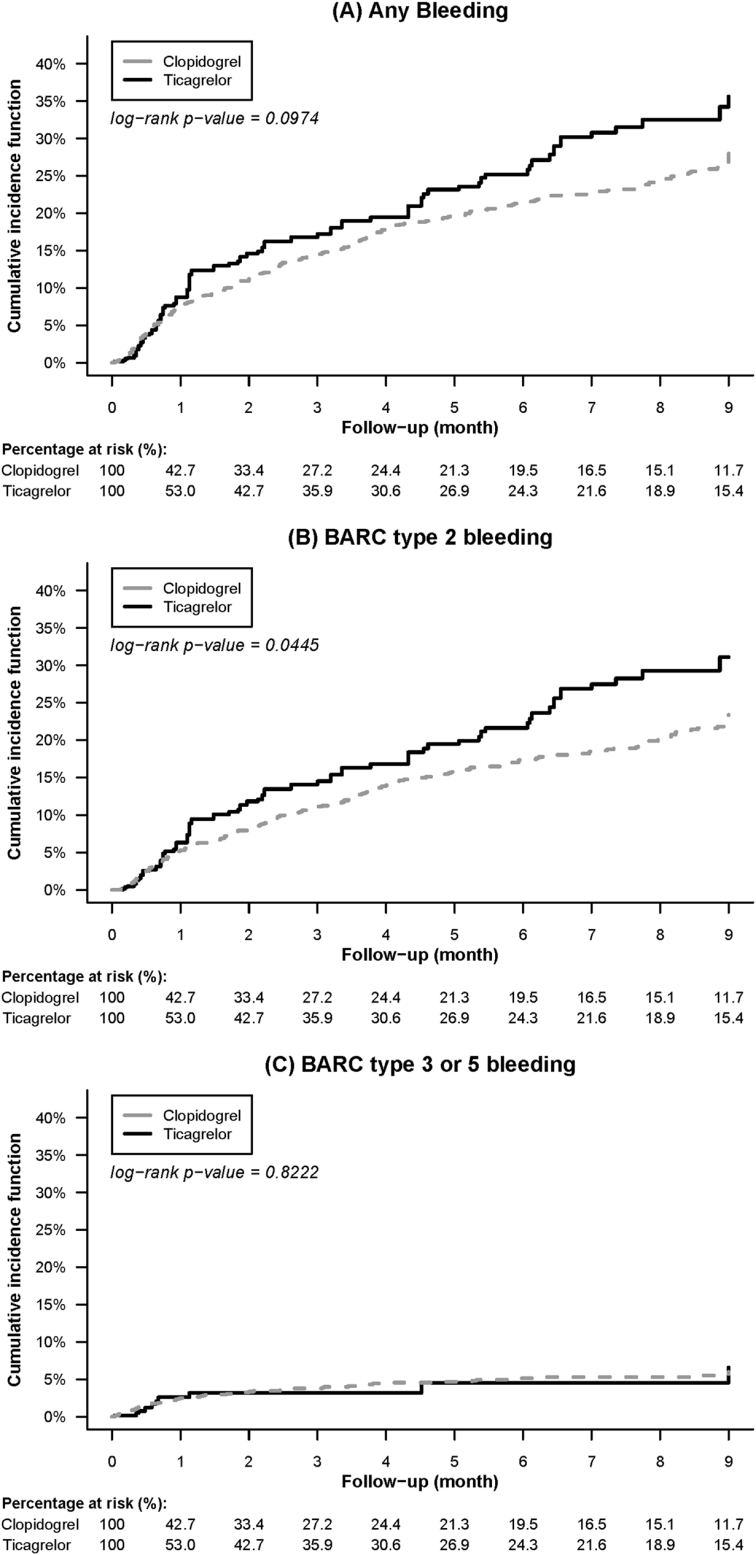
Comparison of the cumulative incidence rates of (**A**) any bleeding, (**B**) BARC type 2, and (**C**) BARC type 3 or 5 bleeding among end-stage renal disease patients with acute myocardial infarction treated between the ticagrelor and clopidogrel groups after stabilized inverse probability of treatment weighting. *BARC* Bleeding Academic Research Consortium.

Figures 2 and 3 illustrate comparisons of the cumulative incidence rates of the efficacy and bleeding endpoints between the two study groups after stabilized IPTW, respectively. Most deaths and BARC type 3 or 5 bleeding occurred during the first month after AMI, whereas BARC type 2 bleeding, the most common type of bleeding, constantly increased over the follow-up period. Supplemental Table 2 lists the efficacy and bleeding endpoints before weighting.

## Discussion

This retrospective nationwide cohort study compared clinical outcomes after ticagrelor versus clopidogrel in combination with aspirin for the treatment of ESRD patients who were diagnosed with AMI. The principal findings of this study are as follows: (1) ticagrelor was comparable to clopidogrel regarding the primary composite outcome of all-cause death, non-fatal MI, or non-fatal stroke; and (2) ticagrelor did not significantly increase the risk of bleeding after accounting for the competing risk of death.

Post hoc analyses of studies evaluating the addition of clopidogrel to aspirin have shown divergent results in the CKD population, with reduced or lack of effect observed with clopidogrel versus placebo^15,30,31^. Clopidogrel resistance, or HPR under clopidogrel therapy, has been recognized to be one of the plausible explanations for increased cardiovascular events in these patients. The prevalence of HPR in the ESRD population is even higher, reaching up to 60% to 80%^32,33^. Therefore, some physicians may consider more effective platelet-inhibiting strategies for selected ESRD patients at high risk for ischemic and thrombotic events. As shown in patients with normal^17^ and impaired kidney function^34^, ticagrelor exhibits potent antiplatelet effects and fast onset of action in those on dialysis compared with clopidogrel, with a prevalence of HPR ranging from 10 to 47%^35,36^. Even though evidence is lacking and bleeding remains a concern, the impetus to prescribe ticagrelor to ESRD patients may be due in part to the remarkable outcomes of the CKD subgroup in the PLATO trial. In a sub-analysis of the PLATO study, ticagrelor was associated with a 4.0% absolute and 28% relative risk reduction of all-cause mortality in CKD patients, and the mortality benefit was even greater in patients with advanced CKD^19^. Consistent with the overall PLATO population, the incidence of non–CABG-related major bleeding was higher in the ticagrelor group than in the clopidogrel group, but this difference did not increase in the CKD cohort. A Swedish database study supported the advantage of ticagrelor in CKD for the real-world treatment of AMI^37^. Across all strata of estimated glomerular filtration rates (eGFRs) in this study, ticagrelor reduced ischemic events but increased bleeding compared with clopidogrel. Notably, ticagrelor was associated with mortality reduction in patients with normal or mildly reduced eGFR, but this benefit was attenuated among those with eGFR < 30 ml/min/m^2^ (HR, 1.08; 95% CI, 0.70–1.49), with a significant interaction between eGFR and ticagrelor (*p* for interaction = 0.04). The lack of mortality benefit with ticagrelor across the eGFR spectrum may be due to an increased risk of bleeding (HR, 1.79; 95% CI, 1.00–3.21) in patients with eGFR < 30 ml/min/m^2^ compared with those with higher eGFR levels (HR 1.1 and 1.13 for eGFR > 60 ml/min/m^2^ and 30–60 ml/min/m^2^, respectively). Severe CKD (eGFR < 30 ml/min/m^2^) is a major criterion for high bleeding risk in the consensus of Academic Research Consortium for High Bleeding Risk^38^. One possible explanation for incremental bleeding associated with worsening CKD is reduced clearance of antithrombotic drugs. Given the adverse impact of bleeding on mortality^39,40^, increased bleeding with ticagrelor therapy may eventually offset its anti-ischemic efficacy as kidney function deteriorates to severe CKD.

In the present study, ticagrelor was comparable to clopidogrel regarding the composite endpoint of all-cause death, MI, or stroke in patients with ESRD. This finding is consistent with the results of our recent study in a general AMI population in Taiwan^41^. Growing evidence shows that the risk–benefit ratio for antithrombotic therapy may be different between Caucasians and East Asians. Two prospective randomized controlled trials conducted in East Asian patients, the PHILO^42^ and the TICAKOREA studies^43^, did not reproduce the superiority of ticagrelor over clopidogrel in the PLATO study. Ticagrelor was even associated with a higher incidence of clinically significant bleeding in the TICAKOREA study. Despite the high prevalence of clopidogrel hypo-responsiveness among East Asian patients^44^, a higher bleeding risk and a lower ischemic risk have been observed in East Asians who are on DAPT after PCI than in Caucasians, a phenomenon commonly referred to as the “East Asian paradox”^45^. As mentioned above, the results of ticagrelor in treating patients with normal or less advanced CKD may not be extrapolated to dialysis patients. As kidney function deteriorates to ESRD, comorbid burden, traditional and nontraditional uremic risk factors, and dialysis-related factors may not only contribute to mortality in these patient but also have a complex interaction with antiplatelet therapy^4,46^. Potential benefits of ischemic reduction with potent platelet inhibition may have been counterbalanced by increased bleeding risk, leading to an overall neutral impact on mortality in our study.

The superiority of ticagrelor over clopidogrel for MI reduction in the PLATO trial was not seen in our ESRD cohort. In the aforementioned Swedish study, the point estimates for MI indicated a lower risk for ticagrelor compared with clopidogrel in all eGFR strata, except in patients with eGFR < 30 ml/min/m^2^, where the CI was wide and crossed the line of unity. These results suggest that the benefit of preventing subsequent MI with ticagrelor may be attenuated as kidney function progressively declines. Evidence has suggested that ticagrelor could be potentially beneficial for stroke prevention in patients with cardiac or cerebrovascular diseases^47,48^. However, we could not draw a conclusion on whether this benefit exists in patients with ESRD, since the stroke events were far less than deaths or bleeding in this study. Further studies are needed to evaluate the role of ticagrelor in stroke prevention in these patients.

The risk of major bleeding in patients with ESRD is estimated to be 20-fold higher than in patients with normal kidney function^49^. Although bleeding definitions and follow-up duration differ between studies, we noted a remarkably high risk of severe (BARC type 3 or 5) bleeding in our ESRD patients (12% per person-years) compared to the risk of BARC type ≥ 3 bleeding in a general, stable CAD population treated with antiplatelet therapy for secondary prevention (0.6% per person-years) or the risk of major bleeding in patients receiving DAPT after PCI (0.4% to 0.8% per person-years)^50,51^. In fact, the majority of our patients may have had a PRECISE-DAPT sore of ≥ 25 (old age, impaired renal function, heighted inflammatory state during AMI, and a high prevalence of anemia or prior bleeding), indicating a high risk for bleeding^52,53^. To avoid bleeding, a short duration of DAPT is generally recommended for these patients. However, a significant portion of our patients received off-label use of ticagrelor (17.7%), suggesting the concerns over high thrombotic risk following AMI. A US registry study reported contraindicated use of antithrombotic agents in 22% of dialysis patients undergoing PCI, leading to an increased risk of major bleeding among these patients^54^. In our study, the risk of overall bleeding or BARC type 2 bleeding associated with ticagrelor was attenuated after taking the competing risk of death into account. Since we excluded the patients who were prescribed no or single antiplatelet therapy (presumably judged as having high bleeding tendency by physicians), we might have selected a population with a relatively low risk of bleeding. This may have skewed the bleeding outcomes in favor of ticagrelor in our study.

In a retrospective study using the United States Renal Data System, Mavrakanas et al. analyzed the outcomes of P2Y_12_ inhibitors in dialysis patients who received DES and were alive at 90 days after stenting^55^. They found that both prasugrel and ticagrelor were comparable to clopidogrel with respect to ischemic outcomes but were associated with a numerically higher incidence of clinically relevant bleeding. Li et al. also used the NHIRD of Taiwan to compared ticagrelor versus clopidogrel in treating dialysis patients with acute coronary syndrome in the same time frame as our study^56^. They found that ticagrelor was associated with higher risks of MACE and major bleeding compared with clopidogrel. Different enrollment criteria and definitions of clinical endpoints may account for the different results between the two studies. Li et al. excluded the patients who died within 30 days of acute coronary syndrome or received P2Y_12_ inhibitors before the index event, leading to a smaller sample size and a relatively low atherosclerotic burden (13% of patients with underlying coronary artery disease and 3% with peripheral artery disease) and bleeding tendency (3% with prior bleeding) in their study population compared with ours. The concomitant use of parenteral antithrombotic agents (unfractionated or low-molecular-weight heparin and glycoprotein IIb/IIIa inhibitors) may have confounded the comparative outcomes between ticagrelor versus clopidogrel but was not taken into account in the study of Li et al. Furthermore, major studies on antiplatelet therapy in patients with acute coronary syndrome have shown a rapid surge in the clinical events during the early period of follow-up^18,57^. Excluding death in the first 30 days in the study of Li et al. makes it difficult to interpret the results. Therefore, our study may be more representative of the real-world treatment of dialysis patients with AMI.

## Limitations

This retrospective database analysis has several inherent limitations. Our study results may have been subject to selection bias. Physicians may have conceivably prescribed ticagrelor to patients with high risk for ischemic events and a low risk for bleeding. However, their exact reasons for choosing between ticagrelor and clopidogrel are not listed in the database. Another source of selection bias in this study is that only the patients who received, or potentially could tolerate, DAPT were enrolled. To mitigate potential differences in baseline bleeding risk, we incorporated past history of bleeding among other covariates as a proxy of bleeding tendency in stabilized IPTW. Certain factors that may have contributed to outcome differences, including the duration and adequacy of dialysis, parameters of CKD-related mineral and bone disorder such as parathyroid hormone, calcium, and phosphate^58^, left ventricular ejection fraction, levels of troponin and hemoglobin, and coronary angiographic findings, were not included in this database analysis. Although we included all the demographic and clinical variables and concomitant medications for stabilized IPTW, we could not mitigate unknown or unmeasured confounding between the study groups. The lack of laboratory data hindered us from calculating the bleeding scores to weigh the trade-off between ischemic and bleeding risks with ticagrelor treatment in this high-risk population. We also could not differentiate between silent bleeding and anemia due to ESRD in this study. Clinical outcomes were identified based on the ICD-9-CM and ICD-10-CM diagnosis codes without adjudication. Although outcomes like mortality, MI, or stroke have been validated in the NHIRD of Taiwan, how the risk of ascertainment bias may have affected the study results was unknown. The lack of causes of deaths in the database impeded a detailed comparison of cardiac and noncardiac deaths between the study groups. We did not analyze BARC types 3 and 5 bleeding separately or classify stroke into ischemic and hemorrhagic subtypes because of the small numbers of these individual events. Adverse effects like dyspnea or bradyarrhythmias related to inhibition of adenosine uptake by ticagrelor could not be addressed with certainty in this database study. In substudies of PLATO, ticagrelor did not affect pulmonary function, and bradycardia or ventricular pauses related to ticagrelor were predominantly asymptomatic and of no apparent clinical consequence^59,60^. However, we cannot fully recognize the clinical impact of these adverse events in patients with ESRD. Lastly, our results could only be applied to East Asian populations and should be validated with prospective randomized trials.

In conclusion, among patients with ESRD who underwent hemodialysis and received DAPT for AMI, ticagrelor was comparable to clopidogrel with regard to the composite endpoint of all-cause death, MI, or stroke. Ticagrelor did not significantly increase the risk of bleeding compared with clopidogrel in hemodialysis patients. Our findings suggest an altered risk–benefit ratio for ticagrelor when kidney function deteriorates from less advanced CKD to ESRD.

## Supplementary Information

Supplementary Tables.

## Data Availability

Data are available from the National Health Insurance Research Database (NHIRD) published by Taiwan National Health Insurance (NHI) Bureau. Due to legal restrictions imposed by the government of Taiwan in relation to the “Personal Information Protection Act”, data cannot be made publicly available.
