# Supplementary material for: Outcomes after ticagrelor versus clopidogrel treatment in end-stage renal disease patients with acute myocardial infarction: a nationwide cohort study
Source: Sci Rep. 2021 Oct 21;11:20826. doi: 10.1038/s41598-021-00360-0 (PMC8531372; doi:10.1038/s41598-021-00360-0)
Supplement: Supplementary file 1 — Supplementary Tables. [file 41598_2021_360_MOESM1_ESM.docx]

**Table S1. Diagnostic codes for comorbidities and outcomes**

|  | **ICD-9-CM codes** | **ICD-10-CM codes** |
| --- | --- | --- |
| Acute myocardial infarction | 410 | I21, I22 |
| NSTEMI | 410.7, 410.9 | I21.4, I22.2 |
| STEMI | 410.0, 410.1, 410.2, 410.3, 410.4, 410.5, 410.6, 410.8 | I21.0, I21.1, I21.2, I21.3, I21.9, I21.A, I22.0, I22.1, I22.8, I22.9 |
| Diabetes mellitus | 250 | E10, E11, E13 |
| Hypertension | 401, 402 | I10-I16 |
| Hyperlipidemia | 272 | E78 |
| Congestive heart failure | 428 | I11.0, I13.0, I13.2, I50 |
| Peripheral artery disease | 440.0, 440.2, 440.3, 440.8, 440.9, 443, 444.0, 444.22, 444.8, 447.8, 447.9 | I70.2-I70.9, I71, I73.9 |
| Gout | 274 | M10, M1A |
| Atrial fibrillation | 427.31 | I48 |
| Stroke | 430-436, 437.1 | I60, I61, I62, I63, G45 |
| Bleeding | 430, 423.0, 423.3, 431, 432, 456.0, 456.20, 459.0, 530.7, 530.82, 531.0, 531.2, 531.4, 531.6, 532.0, 532.2, 532.4, 532.6, 533.0, 533.2, 533.4, 533.6, 534.0, 534.2, 534.4, 534.6, 535.01, 535.11, 535.21, 535.31, 535.41, 535.51, 535.61, 535.71, 537.83, 537.84, 578.0, 562.02, 562.03, 562.12, 562.13, 568.81, 569.3, 569.85, 569.86, 578.1, 578.9, 596.7, 599.7, 719.1, 784.7, 784.8, 786.3 | I31.2, I31.4, I60, I61, I62, I85.01, I85.11, K22.11, K22.6, K25.0, K25.2, K25.4, K25.6, K26.0, K26.2, K266, K27.0, K27.2, K27.4, K27.6, K28.0, K28.2, K28.4, K28.6, K29.01, K29.21, K29.31, K29.41, K29.51, K29.71, K29.81, K31.811, K31.82, K55.21, K57.11, K57.13, K57.31, K57.33, K63.81, K92.0, K62.5, K92.1, K92.2, K66.1, M25.0, R04, R31, R58 |

Abbreviations: ICD-9-CM, International Classification of Diseases, 9th Revision, Clinical Modification; ICD-10-CM, International Classification of Diseases, 10th Revision, Clinical Modification; NSTEMI, non–ST-segment elevation myocardial infarction; STEMI, ST-segment elevation myocardial infarction.

**Table S2. Clinical outcomes of end-stage renal disease patients with acute myocardial infarction treated with ticagrelor versus clopidogrel before stabilized inverse probability of treatment weighting**

| **Endpoints** | **Ticagrelor**  **(n=530)** | | **Clopidogrel**  **(n=2,462)** | | **Cox proportional hazard model** | | **Competing risk analysis** | |
| --- | --- | --- | --- | --- | --- | --- | --- | --- |
|  | **Events** | **Incidence**  **(per 100 PMs)** | **Events** | **Incidence**  **(per 100 PMs)** | **HR (95% CI)** | ***P* value** | **SHR (95% CI)** | ***P* value** |
| Primary efficacy endpoint* | 143 | 10.30 | 695 | 9.46 | 1.04 (0.87-1.24) | 0.6736 | 1.05 (0.87-1.25) | 0.6331 |
| All-cause death | 123 | 7.86 | 611 | 7.38 | 1.00 (0.82-1.21) | 0.9806 |  |  |
| Nonfatal myocardial infarction | 26 | 1.72 | 121 | 1.52 | 1.13 (0.74-1.72) | 0.5786 | 1.09 (0.71-1.65) | 0.6965 |
| Nonfatal stroke | 4 | 0.26 | 55 | 0.67 | 0.37 (0.13-1.02) | 0.0555 | 0.36 (0.13-0.99) | 0.0482 |
| Any bleeding | 66 | 4.75 | 328 | 4.47 | 1.06 (0.82-1.39) | 0.6481 | 1.02 (0.78-1.32) | 0.8988 |
| BARC type 2 bleeding | 56 | 4.03 | 251 | 3.42 | 1.19 (0.89-1.59) | 0.2437 | 1.15 (0.86-1.53) | 0.3569 |
| BARC type 3 or 5 bleeding | 10 | 0.72 | 77 | 1.05 | 0.67 (0.35-1.29) | 0.2314 | 0.65 (0.33-1.25) | 0.1914 |

* Primary efficacy endpoint: a composite of all-cause death, myocardial infarction, or stroke.

Abbreviations: BARC, Bleeding Academic Research Consortium; PM, person-month; CI, confidence interval; HR, hazard ratio; SHR, subdistribution hazard ratio.
